# Hyperoxemia after reperfusion in cardiac arrest patients: a potential dose–response association with 30-day survival

**DOI:** 10.1186/s13054-023-04379-9

**Published:** 2023-03-06

**Authors:** Akil Awad, Per Nordberg, Martin Jonsson, Robin Hofmann, Mattias Ringh, Jacob Hollenberg, Jens Olson, Eva Joelsson-Alm

**Affiliations:** 1 Department of Clinical Science and Education, Center for Resuscitation Sciences, Södersjukhuset, Karolinska Institutet, Stockholm, Sweden; 2 Function Perioperative Medicine and Intensive Care, Karolinska University Hospital, 17176 Stockholm, Sweden; 3 Department of Clinical Science and Education, Södersjukhuset, Karolinska Institutet, Stockholm, Sweden

**Keywords:** Cardiac arrest, Oxygen, Hyperoxemia, Hyperoxia, Hypoxia, Hypoxemia

## Abstract

**Background:**

Hyperoxemia may aggravate reperfusion brain injury after cardiac arrest. The aim of this study was to study the associations between different levels of hyperoxemia in the reperfusion period after cardiac arrest and 30-day survival.

**Methods:**

Nationwide observational study using data from four compulsory Swedish registries. Adult in- and out-of-hospital cardiac arrest patients admitted to an ICU, requiring mechanical ventilation, between January 2010 and March 2021, were included. The partial oxygen pressure (PaO_2_) was collected in a standardized way at ICU admission (± one hour) according to the simplified acute physiology score 3 reflecting the time interval with oxygen treatment from return of spontaneous circulation to ICU admission. Subsequently, patients were divided into groups based on the registered PaO_2_ at ICU admission. Hyperoxemia was categorized into mild (13.4–20 kPa), moderate (20.1–30 kPa) severe (30.1–40 kPa) and extreme (> 40 kPa), and normoxemia as PaO_2_ 8–13.3 kPa. Hypoxemia was defined as PaO_2_ < 8 kPa. Primary outcome was 30-day survival and relative risks (RR) were estimated by multivariable modified Poisson regression.

**Results:**

In total, 9735 patients were included of which 4344 (44.6%) were hyperoxemic at ICU admission. Among these, 2217 were classified as mild, 1091 as moderate, 507 as severe, and 529 as extreme hyperoxemia. Normoxemia was present in 4366 (44.8%) patients and 1025 (10.5%) had hypoxemia. Compared to the normoxemia group, the adjusted RR for 30-day survival in the whole hyperoxemia group was 0.87 (95% CI 0.82–0.91). The corresponding results for the different hyperoxemia subgroups were; mild 0.91 (95% CI 0.85–0.97), moderate 0.88 (95% CI 0.82–0.95), severe 0.79 (95% CI 0.7–0.89), and extreme 0.68 (95% CI 0.58–0.79). Adjusted 30-day survival for the hypoxemia compared to normoxemia group was 0.83 (95% CI 0.74–0.92). Similar associations were seen in both out-of-hospital and in-hospital cardiac arrests.

**Conclusion:**

In this nationwide observational study comprising both in- and out-of-hospital cardiac arrest patients, hyperoxemia at ICU admission was associated with lower 30-day survival.

**Supplementary Information:**

The online version contains supplementary material available at 10.1186/s13054-023-04379-9.

## Introduction

Brain injuries are the leading cause of death and neurological disability after resuscitated cardiac arrest [1, 2]. The pathophysiological mechanisms following cardiac arrest and the post resuscitation phase, are complex, they start within minutes and can last for several days in patients with return of spontaneous circulation (ROSC) [3–5]. By regulating this process, one could possibly reduce its detrimental effect on the neurological outcome.

Oxygen (O_2_) has a double-edged role in several critical conditions associated with ischemia-reperfusion injuries. In the case of cardiac arrest, the global ischemia, within minutes lead to potentially irreversible injuries, in particular in the brain [6]. At the same time, in cases where ROSC is achieved, reperfusion with oxygenated blood will initiate formation of radical oxygen species (ROS) and subsequent lipid perioxidation of the cell membrane, mitochondrial damage, excitotoxicity, loss of ion gradients and finally, structural brain damage [7–14]. This process begins within 15 min of reperfusion and has in experimental studies been accelerated by supranormal levels of O_2_ in arterial blood, so called hyperoxemia [12, 14].

Current international guidelines recommend the use of 100% inspired oxygen during cardiopulmonary resuscitation (CPR) and after ROSC has been restored until the arterial oxygen saturation can be measured reliably, and thereafter to target for normoxia rather than hyperoxia. However, these recommendations are based on low-certainty evidence [6]. Commonly, in real life situations, patients are administered 100% oxygen from the initiation of resuscitation until patients are admitted to the intensive care unit (ICU), which include the transport to hospital and transfers between different units within the hospital such as catherization lab, Xray department and ICU.

Clinical studies investigating hyperoxemia after cardiac arrest with ROSC are heterogenous and have shown varying results [15–17]. For example, there is a wide variation in timing of PaO_2_ measurements wherein some cases only one single PaO_2_-value up to 24 h after ICU arrival has been analysed [18, 19]. Furthermore, many studies have defined hyperoxemia as PaO_2_ > 40 kPa (300 mmHg), which is way above the upper limit for normoxemia, often defined around 13.3 kPa (100 mmHg) [12, 15]. As a specific cut-off limit for potential toxicity of hyperoxemia after cardiac arrest remains to be determined, it may lay anywhere above the normal range values [6, 15]. Several of the previous studies in the field are too small to provide granular information on hyperoxemia in the early reperfusion phase occurring after ROSC until ICU admission. Thus, it remains uncertain how and when increased levels of PaO2 are associated with worse outcomes.

The aim of this large registry study was to study the associations between 30-day survival and different levels of hyperoxemia (defined as mild to extreme) in the vulnerable reperfusion period from ROSC until ICU admission.

## Material and methods

This was a nationwide observational register-based study, performed in Sweden using data from the 1st of January 2010 to the 29th of March 2021. The data was obtained from four registries: the Swedish Intensive Care Registry (SIR), the National Patient Register, the Cause of Death Register and Swedish Registry for Cardiopulmonary Resuscitation. Ethical approval for the study was obtained from by the Swedish Ethical Review Authority (2022-02450-02).

### Patients

We included mechanically ventilated patients who were ≥ 18 years of age and admitted to an ICU with the primary diagnosis of cardiac arrest. The patients were subsequently reported to SIR during the study period. We excluded patients with missing data on PaO_2_. We also excluded patients treated with extracorporeal membrane oxygenation (ECMO).

#### The Swedish Intensive Care Registry (SIR)

The Swedish Intensive Care Registry (SIR), is a national health registry founded in 2001 [20] and contains a large amount of intensive care data such as diagnoses, inpatient care, interventions and patient reported health effects. The registry´s coverage rate has gradually increased over the years, and in 2021 all hospitals in Sweden reported to SIR [21]. The ICU:s are obligated to register the Simplified Acute Physiology Score (SAPS) 3 to SIR. The SAPS 3 include physiological parameters (e.g. the single lowest PaO_2_) ± 1 h from ICU admission and this is the source to the PaO_2_ used in our study. The registry has previously been described in more detail [21].

#### The National Patient Register (NPR) and Cause of Death Register (CDR)

The NPR and the CDR are maintained by Swedish National Board of Health and Welfare and collect individual patient data including primary cause of admission, co-morbidities, health care episodes and date of death. Data collection is based on social security numbers assigned to all registered residents in Sweden. The NPR includes all in-patient care in Sweden since 1987, the register has been validated several times and the completeness of the reported data is almost 100% [22]. Data on Charlson comorbidity index (CCI) in our study was obtained from the NPR and data on 30-day survival was obtained from the CDR.

#### Swedish Registry for Cardiopulmonary Resuscitation (SRCR)

The SRCR describes both out-of-hospital cardiac arrest (OHCA) and in-hospital cardiac arrest (IHCA) in Sweden. The registry started 1990 for OHCA and 2005 for IHCA and includes patients in whom CPR was started by medical staff [23]. It contains data on interventions, pharmaceuticals, inpatient care and is reported in accordance with the Utstein guidelines [24]. The registry´s degree of coverage has gradually increased over the years, and in 2021 all EMS providers and hospitals in Sweden reported to the registry [23]. The registry has been validated and previously been described in more detail [25, 26].

### Definitions

Patients were divided into groups based on lowest PaO_2_ within one hour from ICU arrival. Hyperoxemia was defined as PaO_2_ > 13.3 kPa (> 100 mmHg), normoxemia as PaO_2_ 8–13.3 kPa (60–100 mmHg) and hypoxemia as PaO_2_ < 8 kPa (< 60 mmHg). Furthermore, patients with hyperoxemia were divided into four groups based on their PaO_2_-level: mild hyperoxemia; 13.4–20 kPa (100–150 mmHg), moderate hyperoxemia; 20.1–30 kPa (150–225 mmHg), severe hyperoxemia; 30.1–40 kPa (225–300 mmHg), and extreme hyperoxemia; > 40 kPa (> 300 mmHg).

The definition of hypoxemia and hyperoxemia was the same as applied in several previous studies on the subject [27–31]. The different thresholds within the hyperoxemia group were pragmatically chosen. The highest threshold (> 40 kPa) within the hyperoxemia group is in accordance with the definition used for hyperoxemia in several previous studies [15, 19, 31].

### Outcomes

The primary outcome was 30-day survival. The main population included all cardiac arrest patients, subgroup populations included patients with OHCA and IHCA, respectively. Subgroup analysis was also performed based on initial rhythm. Furthermore, we analyzed the exposure of PaO_2_ as a continuous variable to explore a possible cut-off level for worse outcome.

### Statistical analysis

Normally distributed continuous variables are presented as means and standard deviations, non-normally distributed continuous variables are presented as medians and quartiles, and categorical variables are presented as counts and proportions. Differences in baseline characteristics was assessed with standardized mean difference (SMD). The normoxia group (PaO_2_ 8–13.3) was used as a reference group and the other groups were compared with the normoxia group. To estimate the relative risk (RR) and 95% confidence interval (CI) multivariate modified Poisson regression analysis was performed. Adjustment for age, sex, initial shockable rhythm, bystander CPR, witnessed status, location, EMS/Rapid response team-response time, Charlson comorbidity index (CCI) and SAPS-3-score was made. *P*-values < 0.05 was considered significant. As a sensitivity analysis, the relationship between arterial oxygen levels and survival was analysed using multiple logistic regression with b-splines, with 4 degrees of freedom, for the continuous PaO_2_ variable. Missing data was handled using multiple imputation by chained equations (mice) under the assumption of missing at random. Fifteen datasets were created and the results from the main analyses were pooled using Rubins´s rule. As a sensitivity analysis, the regression models were also performed on complete cases. The statistical software R version 4.1.3 (R foundation for Statistical Computing, Vienna, Austria) was used for all analyses.

## Results

A total of 12833 adult cardiac arrest patients were identified in the registries for the study period, of which 9735 fulfilled the inclusion criteria and were included in the analysis.

**Fig. 1:**
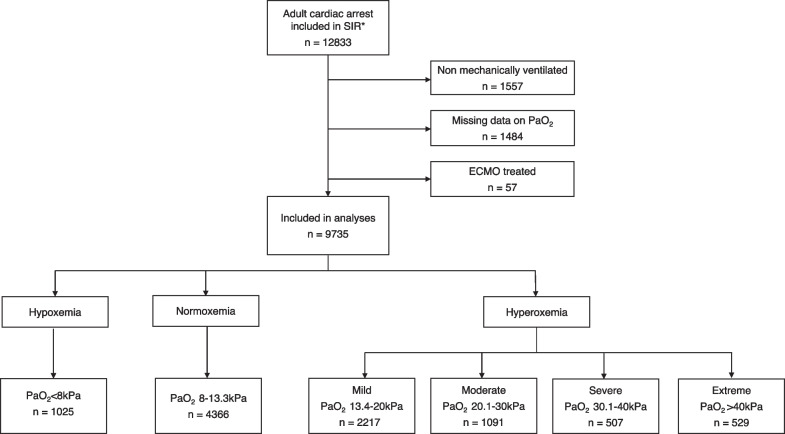
Flow of cardiac arrest patients. *Swedish intensive care registry

Of those, 4344 (44.6%) patients were classified as hyperoxemia, 4366 (44.8%) patients as normoxemia and 1025 (10.5%) patients as hypoxemia. Within the hyperoxemia group, 2217 patients had mild hyperoxemia (PaO_2_ 13.4–20 kPa), 1091 patients had moderate hyperoxemia (PaO_2_ 20.1–30 kPa), 507 patients had severe hyperoxemia (PaO_2_ 30.1–40 kPa) and 529 patients had extreme hyperoxemia (PaO_2_ > 40 kPa). Flow of patients can be seen in Fig. 1 and the distribution of patients based on PaO_2_ can be seen in Additional file 6: Fig. 6. Furthermore, 6202 OHCA patients and 3533 IHCA patients were included in the analysis (Additional file 1: Fig. 1 and Additional file 2: Fig. 2).

Baseline characteristics for the included patients are shown in Table 1. Most characteristics were similar between the groups, e.g. the time from cardiac arrest to ICU arrival; 94–114 min for OHCA patients and 35–43 min for IHCA patients. The hypoxemia group had slightly higher SAPS3-score and somewhat fewer patients in the hypoxemia and PaO_2_ > 40 kPa-group had an initial shockable rhythm. The physiological parameters ± 1 h from ICU admission for the study patients are presented in Table 2.

**Table 1:** Baseline Characteristics of the study population

	Hypoxemia	Normoxemia	Hyperoxemia				SMD^**¶**^	Missing, %
*All Cardiac arrests*								
PaO_2_	< 8 kPa	8–13.3 kPa	13.4–20 kPa	20.1–30 kPa	30.1–40 kPa	> 40 kPa		
*n* (%)	1025 (11)	4366(45)	2217 (23)	1091 (11)	507 (5)	529 (5)		
Age, median (IQR), y	70 (59–77)	70 (61–77)	68 (57–77)	68 (57- 76)	68 (56–77)	70 (59–78)	0.09	0
Women, no (%)	377 (37)	1462 (34)	697 (32)	357 (33)	166 (33)	202 (38)	0.07	0.1
Witnessed cardiac arrest, no (%)	827 (82)	3537 (83)	1688 (78)	813 (77)	374 (76)	400 (78)	0.09	2.3
Shockable rhythm, no (%)	249 (29)	1435 (39)	786 (41)	351 (37)	178 (40)	132 (29)	0.13	14.8
SAPS3^#^-score, median (IQR)	85 (76–94)	79 (69–88)	77 (68–86)	78 (68–86)	78 (70–86)	79 (71–87)	0.23	0
CCIº, mean (SD)	2.6 (2.2)	2.7 (2.3)	2.5 (2.2)	2.4 (2.2)	2.3 (2.1)	2.4 (2.3)	0.08	0
Median time from cardiac arrest to ICU arrival, min (IQR)	70 (37–119)	88 (48–143)	93 (53–147)	89 (58–132)	89 (53–131)	84 (54–121)	0.127	7.5
*OHCA*								
PaO_2_	< 8 kPa	8–13.3 kPa	13.4–20 kPa	20.1–30 kPa	30.1–40 kPa	> 40 kPa		
n (%)	512 (8)	2664 (43)	1522 (25)	783 (13)	349 (6)	372 (6)		
Age, median (IQR), y	69 (56–77)	68 (58–76)	67 (56–76)	67 (55–76)	66 (55–76)	69 (57–77)	0.08	0
Women, no (%)	162 (32)	807 (30)	449 (30)	230 (29)	108 (31)	141 (38)	0.07	0.1
Median EMS* response time, min (IQR)	8 (5.0–13)	7 (5–11)	7 (5–11)	7.0 (5–11)	7 (5–11)	7 (5–12)	0.07	3.1
Location at home, no (%)	317 (62)	1665 (63)	925 (61)	472 (60)	232 (67)	269 (73)	0.13	0.3
Witnessed cardiac arrest, no (%)	397 (79)	2081 (80)	1116 (75)	567 (75)	249 (74)	274 (77)	0.07	2.8
Bystander CPR, no (%)	305 (63)	1586 (62)	928 (64)	437 (58)	194 (58)	205 (58)	0.07	4.3
Shockable rhythm, no (%)	183 (40)	1127 (48)	659 (48)	303 (43)	145 (46)	108 (33)	0.14	11
SAPS3-score, median (IQR)	82 (75–90)	77 (68–86)	75 (67–84)	76 (67–85)	77 (69–84)	79 (70–85)	0.23	0
CCI, mean (SD)	2.3 (2.1)	2.3 (2.2)	2.2 (2.1)	2.0 (2.0)	2.1 (2.2)	2.1 (2.1)	0.07	0
Median time from cardiac arrest to ICU arrival, min (IQR)	105 (73–142)	114 (79–167)	111(77–163)	101 (75–150)	98 (77–148)	94 (73–132)	0.14	1.6
*IHCA*								
PaO_2_	< 8 kPa	8–13.3 kPa	13.4–20 kPa	20.1–30 kPa	30.1–40 kPa	> 40 kPa		
*n* (%)	513 (15)	1702 (48)	695 (20)	308 (9)	158 (4)	157(4)		
Age, median (IQR), y	72 (62–78)	72 (64–78)	70 (62–78)	72 (63–78)	70 (59–77)	72 (63–79)	0.10	0
Women, no (%)	215 (42)	655 (39)	248 (36)	127 (41)	58 (37)	61 (39)	0.06	0
Median RRT^¥^-response time, min (IQR)	3 (2–5)	3 (2–5)	3 (2–5)	3 (2–5)	3 (2–5)	3 (2–5)	0.04	26.5
Location at general ward, no (%)	261 (52)	820 (49)	337 (51)	166 (56)	88 (56)	85 (56)	0.28	2.9
Witnessed cardiac arrest, no (%)	430 (85)	1456 (87)	572 (84)	246 (81)	125 (80)	126 (82)	0.08	1.5
Bystander CPR, no (%)	419 (94)	1413 (93)	577 (93)	246 (90)	127 (89)	131 (93)	0.09	10.8
Shockable rhythm, no (%)	66 (17)	308 (23)	127 (23)	48 (20)	33 (25)	24 (19)	0.09	21.4
SAPS3-score, median (IQR)	88 (79–96)	83 (72–93)	80 (70–90)	82 (75–91)	83 (74–93)	82 (73–91)	0.19	0
CCI, mean (SD)	2.9 (2.3)	3.2 (2.4)	3.1 (2.5)	3.2 (2.3)	2.8 (2.0)	3.1 (2.7)	0.1	0
Median time from cardiac arrest to ICU arrival, min (IQR)	37 (23–56)	40 (25–66)	43 (28–70)	43 (27–71)	36 (24–60)	35 (26–53)	0.08	17.7

^¥^Rapid response team

^*^Emergency medical services

^#^Simplified acute physiology score 3

ºCharlson comorbidity index

^¶^Standard mean difference

**Table 2:** Physiological parameters at ICU admission (± one hour)

	Hypoxemia	Normoxemia	Hyperoxemia				SMD^¶^	Missing, %
*All cardiac arrest*								
PaO_2_	< 8 kPa	8–13.3 kPa	13.4–20 kPa	20.1–30 kPa	30.1–40 kPa	> 40 kPa		
*n*	1025	4366	2217	1091	507	529		
PaO_2_ (lowest), median (IQR)	6.8 (5.9–7.4)	10.5 (9.3–11.9)	16 (14.5–17.9)	24 (22–26.8)	34.7 (32.2–37)	52 (45.8–61)	5.778	0
Fraction inspired O_2_ (%),mean (SD)	81 (23)	66 (23)	62 (23)	63 (23)	71 (23)	85 (21)	0.51	1.7
Systolic blood pressure (lowest), mean (SD)	63(41)	74 (41)	77 (42)	78 (42)	74 (43)	69 (43)	0.16	1.3
Heart rate (highest), mean (SD)	96 (41)	95 (36)	92 (36)	94 (35)	93 (34)	92 (36)	0.06	1.2
GCS (lowest), median (IQR)	3 (3–4)	3 (3–6)	3 (3–6)	3 (3–5)	3 (3–4)	3 (3–3)	0.14	70
Highest temperature (C^o^), mean (SD)	35.6 (2.1)	35.7 (1.5)	35.6 (1.4)	35.5 (1.4)	35.4 (1.5)	35.3 (2.1)	0.13	6.4
Arterial pH (lowest), mean (SD)	7.1 (0.2)	7.2 (0.2)	7.2 (0.2)	7.2 (0.2)	7.2 (0.2)	7.1 (0.2)	0.15	1.1
Bilirubine (highest), mean (SD)	14 (26)	13 (25)	13 (22)	13 (32)	12 (18)	12 (16)	0.04	12
*OHCA*								
PaO_2_	< 8 kPa	8–13.3 kPa	13.4–20 kPa	20.1–30 kPa	30.1–40 kPa	> 40 kPa		
*n*	512	2664	1522	783	349	372		
PaO_2_ (lowest), median (IQR)	6.9 (6.0–7.5)	10.7 (9.4–12)	16.0 (14.5–17.9)	24 (21.9–26.8)	34.6 (32.4–37)	52 (45.9–60)	5.8	0
Fraction inspired O_2_ (%), mean (SD)	78 (23)	63 (23)	59 (22)	60 (22)	69 (23)	84 (23)	0.55	1.7
Systolic blood pressure (lowest), mean (SD)	70 (41)	79 (40)	80 (41)	83 (41)	80 (41)	73 (42)	0.15	1.1
Heart rate (highest), mean (SD)	95 (37)	91 (33)	88 (32)	93 (32)	92 (30)	89 (32)	0.09	1.1
GCS (lowest), median (IQR)	3 (3–4)	3 (3–5)	3 (3–4)	3 (3–4)	3 (3–3)	3.0 (3–3)	0.14	68.2
Highest temperature (C^o^), mean (SD)	35.1 (2.3)	35.5 (1.5)	35.4 (1.4)	35.3 (1.4)	35.1 (1.4)	35.1 (2.3)	0.12	5.2
Arterial pH (lowest), mean (SD)	7.1 (0.2)	7.2 (0.2)	7.2 (0.2)	7.2 (0.2)	7.2 (0.2)	7.1 (0.2)	0.16	1
Bilirubine (highest), mean (SD)	11 (11)	11 (23)	11 (16)	11 (20)	11 (12)	11 (16)	0.02	11.3
*IHCA*								
PaO_2_	< 8 kPa	8–13.3 kPa	13.4–20 kPa	20.1–30 kPa	30.1–40 kPa	> 40 kPa		
*n*	513	1702	695	308	158	157		
PaO_2_ (lowest), median (IQR)	6.7 (5.9–7.4)	10.3 (9.1–11.8)	15.9 (14.5–17.8)	24 (22.1–26.6)	34.8 (32–36.9)	52.6 (45–63)	5.7	0
Fraction inspired O_2_ (%), mean (SD	84 (22)	71 (23)	69 (24)	71 (23)	77 (21)	87 (20)	0.41	1.7
Systolic blood pressure (lowest), mean (SD)	57 (40)	66 (42)	69 (43)	66 (42)	62 (45)	59 (43)	0.13	1.6
Heart rate (highest), mean (SD)	99 (46)	100 (41)	99 (41)	96 (41)	97 (42)	100 (44)	0.05	1.3
GCS (lowest), median (IQR)	3 (3–5)	3 (3–9)	3 (3–9)	3 (3–7)	3 (3–4)	3 (3–8)	0.21	71.9
Highest temperature (C^o^), mean (SD)	36.2 (1.6)	36.2 (1.4)	36.2 (1.3)	36.1 (1.3)	36 (1.5)	35.8 (1.6)	0.14	8.4
Arterial pH (lowest), mean (SD)	7.1 (0.2)	7.2 (0.2)	7.2 (0.2)	7.2 (0.2)	7.2 (0.2)	7.1 (0.2)	0.14	1.3
Bilirubine (highest), mean (SD)	18 (35)	15 (27)	16 (30)	20 (50)	15 (27)	14 (16)	0.07	13.9

### Outcome

**Fig. 2:**
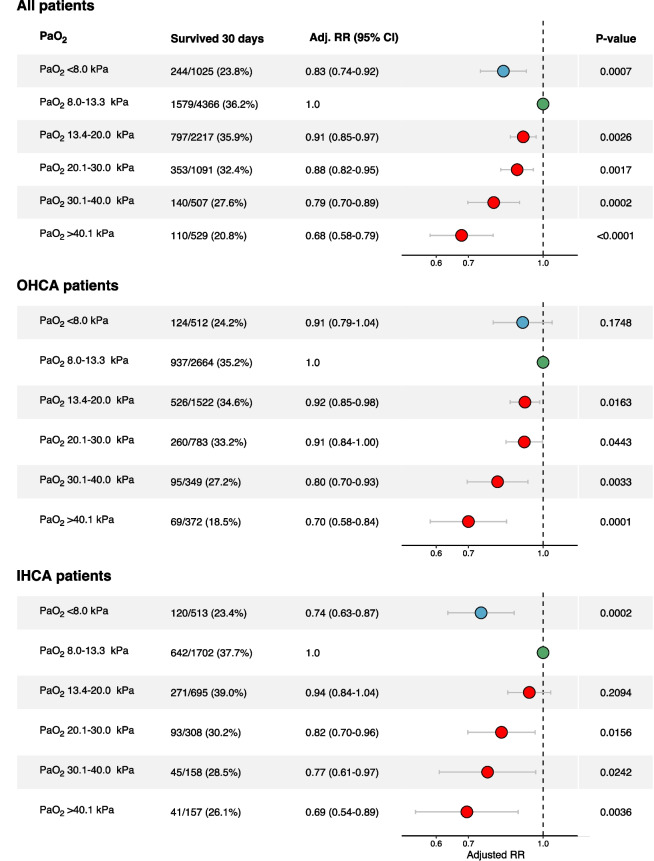
Adjusted RR for 30-day survival in different O_2_-groups compared to normoxemia group. Adjusted for sex, age, witnessed status, bystander CPR, location, EMS/Rapid response team-response time, initial rhythm, Charlson comorbidity index, SAPS 3 score

**Fig. 3:**
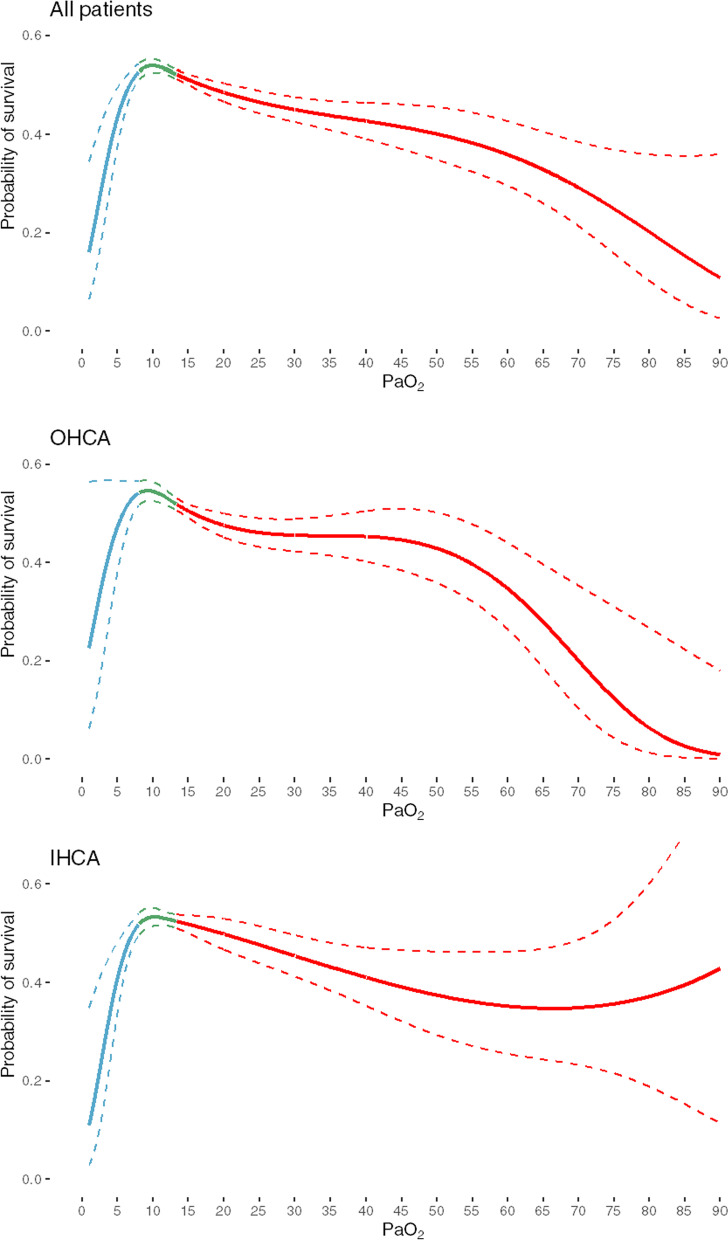
Adjusted predicted 30-day survival with PaO_2_ as a continuous variable (b-splines with 4 degrees of freedom). Adjusted for sex, age, witnessed status, bystander CPR, location, EMS/Rapid response team-response time, initial rhythm, Charlson comorbidity index, SAPS 3 score. Dotted lines represent 95% confidence intervals

Patient outcomes are presented in Fig. 2 and in Additional file 7: Fig. 7. The crude 30-day survival rates were 32.2% in the hyperoxemia group, 36.2% in the normoxemia group and 23.8% in the hypoxemia group. Compared to the normoxemia group, the adjusted RR for 30-day survival in the whole hyperoxemia group was 0.87 (95% CI 0.82–0.91). Among patients with hyperoxemia, survival was successively (p-value for trend < 0.0001) decreasing with higher PaO_2_-levels (Figs. 2 and 3). Compared to the normoxemia group, the adjusted RR for the mild hyperoxemia group was 0.91 (95% CI 0.85–0.97), for the moderate hyperoxemia group 0.88 (95% CI 0.82–0.95), for the severe hyperoxemia group 0.79 (95% CI 0.7–0.89), and for the extreme hyperoxemia group 0.68 (95% CI 0.58–0.79). Adjusted RR for the hypoxemia compared to normothermia group was 0.83 (95% CI 0.74–0.92). A similar pattern was seen in OHCA and IHCA (Fig. 2). The adjusted 30-day survival with PaO_2_ as continuous variable is presented in Fig. 3.

Further subgroup analyses with regard to first cardiac rhythm are presented in Additional file 4: Fig. 4 and Additional file 5: Fig. 5. Similar associations were found, but strongest among patients with initial non-shockable rhythms.

### Sensitivity analysis

Complete cases yielded similar results (Additional file 3: Fig. 3) as the main (imputed) data set.

## Discussion

The main finding of this nationwide study of resuscitated cardiac arrest patients was that hyperoxemia compared to normoxemia at the time of ICU admission was associated with worse outcomes, this was most apparent in the groups with severe and extreme hyperoxemia. This is new information derived from an unselected, all-comer population and has not been seen in previous observational studies. In addition, the same potential “dose-response” associations were seen in both OHCA and IHCA patients. As expected, hypoxemia at ICU admission was associated with lower 30-day survival compared to normoxia.

Following cardiac arrest, the cerebral tissue oxygen tension (PbtO_2_) drops within minutes to almost zero [32, 33]. By administration of a high fraction of inspired oxygen (FiO_2_) during CPR, the PbtO_2_ during optimal experimental conditions, can be restored close to pre-arrest levels [32–34]. Furthermore, observational studies have demonstrated an association between higher PaO_2_ during CPR and greater probability to obtain ROSC [35, 36]. On the other hand, at time of ROSC in real life cases, the PaO_2_ is changed from commonly severe hypoxemia to oxygenated reperfusion with continued high O_2_ delivery. Subsequently, the PbtO_2_ increases severalfold [32, 33] and experimental and observational data suggest that this hyperoxemia could be harmful during the reperfusion phase [7–9, 37]. The fact that the effects of reperfusion, and the risk for formation of ROS, increases dramatically within minutes after ROSC, may be a possible physiological explanation for our findings.

According to current European guidelines, 100% O_2_ should be given during CPR and in case of ROSC one should aim for arterial blood oxygen saturation 94–98% as soon as this can be monitored safely [6]. These recommendations, however, are based on a low degree of evidence and are difficult to follow in a controlled way in the early post-ROSC period that is associated with ambulance transport, admission to the emergency department, intra-hospital transfer and, finally, admission to the ICU which in this cohort occurred in median about two hours after the cardiac arrest in the OHCA patients. Previous observational studies have heterogenous study designs, measure oxygen levels at different time points and show inconsistent results [15, 18, 28, 38]. In two landmark studies published more than 10 years ago, only one single PaO_2_-value up to 24 h from ICU admission was analyzed [18, 19]. A recent meta-analysis concluded that most observational studies comparing PaO_2_-levels after ROSC suffer from a high risk of bias and therefore pooled analysis was not performed [15]. Most studies did not reach statistical significance, but most of the individual studies had point estimates favouring normoxemia over hyper-/hypoxemia.

We believe that our findings are based on measurements of PaO_2_ at an important time point within a few hours from reperfusion after ROSC and support current guideline recommendation to strive for normoxemia as soon ROSC is achieved. To the best of our knowledge, this is the first time such a potential dose-response relationship between early PaO_2_-levels after ROSC and survival has been demonstrated in a real-world clinical setting. A dose-response relationship increases the likelihood for an underlying biological mechanism. Nevertheless, despite our large cohort, it is difficult to determine an exact cut-off point for when hyperoxemia becomes associated with worse outcome. However, instead of looking for an arbitrary cut-off point, it might be reasonable to regard supranormal PaO_2_ as a continuum: the higher, the more dangerous. This approach is supported by the appearance of the survival curves with PaO_2_ as continuous variable (Fig. 3).

The results from this study need to be put in the light of previous studies. Several RCT:s have studied the effect of different oxygen therapy strategies in ICU patients without demonstrating any significant difference in clinically relevant outcomes [16, 39–42]. Of notice, most of these studies did not specifically study cardiac arrest patients and in all of these studies, the “liberal oxygen groups” had PaO_2_-levels that correspond to the lower range in the mild hyperoxia group in our study. It is reasonable to believe that the level of hyperoxemia in these studies was too modest and/or too late to cause harm. In the recently published EXACT-trial, that randomized lower (SpO_2_ 90–94%) vs higher (SpO_2_ 99–100%) O_2_ saturation targets in resuscitated cardiac arrest patients in the prehospital setting, the lower SpO_2_ -target group had more hypoxic events and a trend towards lower survival [17] which is in agreement with our findings. The median first PaO_2_ in ICU in the higher SpO_2_ -target group was 15.2 kPa (114 mmHg), i.e. corresponding to the lower range of the modest hyperoxemia group in our study. Thus, these findings are not applicable to our data from an unselected population where a substantial proportion of patients had significantly higher PaO_2_-levels.

A prospective cohort study from Roberts et al. [27] showed that early hyperoxemia was independently associated with worse neurological function at hospital discharge. The PaO_2_-cut point for worse outcome started at ≥ 40 kPa. In a recently published sub-analysis of the Target Temperature Management 2 (TTM2) trial, both hypoxemia and severe hyperoxemia were significantly associated with 6 month-mortality [43]. The best cut-off point associated with 6 month-mortality was 195 mmHg (26 kPa), which resembles the results in our study. This association could not be seen in a post hoc analysis of the first Targeted Temperature Management (TTM) trial [29]. All the above studies focused on PaO_2_-levels after ICU-arrival, while our study focuses more on the initial phase after the cardiac arrest including only samples collected within one hour from ICU admission. In a Finish study, including 1110 patients, there was no association between early hyperoxemia (on the first PaO_2_ collected after ROSC) and favorable neurological outcome [30].

Oxygen therapy after cardiac arrest has become a major research question in post resuscitation management [44]. In practice, it may be difficult to titrate oxygen saturation in a controlled and safe manner in the prehospital setting, which was recently highlighted in the EXACT trial [17]. Most often, there is no possibility to analyze arterial blood gases and measurement of oxygen saturation by pulse oximetry (SpO_2_) may be unreliable due to for example vasoconstriction. In a study by Young et al. [45] where participants were randomized to either targe SpO_2_ 90–94% or SpO_2_ 100%, only 18 patients were enrolled before the trial was stopped due to safety concerns (7/8 patients in lower target group had an episode of SpO_2_ < 88%). Fortunately, there are currently several registered RCT:s investigating optimal oxygenation targets at different time points after cardiac arrest which may aid to close this important gap of knowledge [15].

This study has several strengths and adds novel information to this clinically important research question. Our national registers are of high quality and include most of the patients who were treated in an ICU after cardiac arrest in Sweden during the study period. The proportion of missing data is low, for the exposure (PaO_2_) values were missing for approximately 15% of patients and for the main outcome (mortality) we have complete data. All registered blood gas results were collected from one hour before to one hour after admission to the ICU, thus comparatively in the early reperfusion period after the cardiac arrest and within the period where most of the cellular injuries occur. Thanks to the large study population, we were able to perform more granular analyses than most other studies on the subject.

However, there are several limitations. First, we know only the registered PaO_2_ is ± 1 h from ICU arrival, but the exact time point is unknown. Second, we only have data on the lowest registered PaO_2_ and therefore there may be patients who initially had even higher PaO_2_ levels at an earlier time point that subsequently were titrated down, thus leading to misclassification. However, if anything, it strengthens the likelihood that those classified as severe hyperoxemia have been exposed to high oxygen levels more than just briefly. Third, our registries only contain one registered PaO_2_ per patient and therefore we were unable to analyse the effect of hyperoxemia over time. Fourth, since it is an observational study one can only draw conclusions about association and not causality. Although we tried to adjust for known confounders there is always a risk of residual unmeasured confounding. Since we have studied the association between a blood value (PaO_2_) and outcome, and not a treatment strategy per se, one should be careful when drawing conclusions. Fifth, long-term neurological function is an important outcome after cardiac arrest that we were not able to measure in this study.

## Conclusion

In this nationwide observational study comprising both in- and out-of-hospital cardiac arrest patients, hyperoxemia at ICU admission was + associated with lower 30-day survival.

### Supplementary Information

**Additional file 1**. **Supplementary Figure 1**. Flow of OHCA patients.

**Additional file 2**. **Supplementary Figure 2**. Flow of IHCA patients.

**Additional file 3**. **Supplementary Figure 3**. Adjusted RR for 30-day survival in complete cases.

**Additional file 4**. **Supplementary Figure 4**. Adjusted RR for 30-day survival in patients with shockable rhythms.

**Additional file 5**. **Supplementary Figure 5**. Adjusted RR for 30-day survival in patients with non-shockable rhythms.

**Additional file 6**. **Supplementary Figure 6**. Distribution of patients based on PaO_2_

**Additional file 7**. **Supplementary Figure 7**. Kaplan-Meier survival analysis.

## Data Availability

The data used and analyzed in this study is not publicly available but is accessible from the lead author on reasonable request.

## References

[1] Nolan JP, Soar J, Cariou A, Cronberg T, Moulaert VR, Deakin CD (2015). European resuscitation council and European society of intensive care medicine guidelines for post-resuscitation care 2015: section 5 of the European resuscitation council guidelines for resuscitation 2015. Resuscitation..

[2] Lemiale V, Dumas F, Mongardon N, Giovanetti O, Charpentier J, Chiche JD (2013). Intensive care unit mortality after cardiac arrest: the relative contribution of shock and brain injury in a large cohort. Intensive Care Med.

[3] Froehler MT, Geocadin RG (2007). Hypothermia for neuroprotection after cardiac arrest: mechanisms, clinical trials and patient care. J Neurol Sci.

[4] Nolan JP, Neumar RW, Adrie C, Aibiki M, Berg RA, Bottiger BW (2008). Post-cardiac arrest syndrome: epidemiology, pathophysiology, treatment, and prognostication. A scientific statement from the International Liaison Committee on Resuscitation; the American Heart Association Emergency Cardiovascular Care Committee; the Council on Cardiovascular Surgery and Anesthesia; the Council on Cardiopulmonary, Perioperative, and Critical Care; the Council on clinical cardiology; the Council on stroke. Resuscitation.

[5] Adrie C, Adib-Conquy M, Laurent I, Monchi M, Vinsonneau C, Fitting C (2002). Successful cardiopulmonary resuscitation after cardiac arrest as a "sepsis-like" syndrome. Circulation.

[6] Nolan JP, Sandroni C, Böttiger BW, Cariou A, Cronberg T, Friberg H (2021). European resuscitation council and European society of intensive care medicine guidelines 2021: post-resuscitation care. Intensive Care Med.

[7] Dell'Anna AM, Lamanna I, Vincent JL, Taccone FS (2014). How much oxygen in adult cardiac arrest?. Crit Care.

[8] Hazelton JL, Balan I, Elmer GI, Kristian T, Rosenthal RE, Krause G (2010). Hyperoxic reperfusion after global cerebral ischemia promotes inflammation and long-term hippocampal neuronal death. J Neurotrauma.

[9] Pilcher J, Weatherall M, Shirtcliffe P, Bellomo R, Young P, Beasley R (2012). The effect of hyperoxia following cardiac arrest—A systematic review and meta-analysis of animal trials. Resuscitation.

[10] Watson NA, Beards SC, Altaf N, Kassner A, Jackson A (2000). The effect of hyperoxia on cerebral blood flow: a study in healthy volunteers using magnetic resonance phase-contrast angiography. Eur J Anaesthesiol.

[11] Floyd TF, Clark JM, Gelfand R, Detre JA, Ratcliffe S, Guvakov D (2003). Independent cerebral vasoconstrictive effects of hyperoxia and accompanying arterial hypocapnia at 1 ATA. J Appl Physiol.

[12] Singer M, Young PJ, Laffey JG, Asfar P, Taccone FS, Skrifvars MB (2021). Dangers of hyperoxia. Crit Care.

[13] Quintard H, Patet C, Suys T, Marques-Vidal P, Oddo M (2015). Normobaric hyperoxia is associated with increased cerebral excitotoxicity after severe traumatic brain injury. Neurocrit Care.

[14] Alva R, Mirza M, Baiton A, Lazuran L, Samokysh L, Bobinski A (2022). Oxygen toxicity: cellular mechanisms in normobaric hyperoxia. Cell Biol Toxicol.

[15] Holmberg MJ, Nicholson T, Nolan JP, Schexnayder S, Reynolds J, Nation K (2020). Oxygenation and ventilation targets after cardiac arrest: a systematic review and meta-analysis. Resuscitation.

[16] Schmidt H, Kjaergaard J, Hassager C, Mølstrøm S, Grand J, Borregaard B (2022). Oxygen targets in comatose survivors of cardiac arrest. N Engl J Med.

[17] Bernard SA, Bray JE, Smith K, Stephenson M, Finn J, Grantham H (2022). Effect of lower vs. higher oxygen saturation targets on survival to hospital discharge among patients resuscitated after out-of-hospital cardiac arrest: the EXACT randomized clinical trial. JAMA.

[18] Bellomo R, Bailey M, Eastwood GM, Nichol A, Pilcher D, Hart GK (2011). Arterial hyperoxia and in-hospital mortality after resuscitation from cardiac arrest. Crit Care.

[19] Kilgannon JH, Jones AE, Shapiro NI, Angelos MG, Milcarek B, Hunter K (2010). Association between arterial hyperoxia following resuscitation from cardiac arrest and in-hospital mortality. JAMA.

[20] SIR. [updated 2021-08-12. Available from https://www.icuregswe.org/en/about-sir/organization/.

[21] SIR. Årsrapport 2021 [updated 2022-03-17. Available from: https://www.icuregswe.org/globalassets/arsrapporter/arsrapport_2021.pdf.

[22] Patientregistret. [updated 2019/05/20. Available from: https://www.socialstyrelsen.se/en/statistics-and-data/registers/register-information/the-national-patient-register/.

[23] HLR-rådet. HLR-registret årsrapport 2021 2022 [Available from: https://registercentrum.blob.core.windows.net/shlr/r/SHLR-rsrapport-med-data-fr-n-2021-B1x0F0cFGs.pdf.

[24] Langhelle A, Nolan J, Herlitz J, Castren M, Wenzel V, Soreide E (2005). Recommended guidelines for reviewing, reporting, and conducting research on post-resuscitation care: the Utstein style. Resuscitation.

[25] Stromsoe A, Svensson L, Axelsson AB, Goransson K, Todorova L, Herlitz J (2013). Validity of reported data in the Swedish cardiac arrest register in selected parts in Sweden. Resuscitation.

[26] HLR-registret. [updated 2020/07/01. Available from: http://kvalitetsregister.se/englishpages/findaregistry/registerarkivenglish/nationalqualityregistryforcardiopulmonaryresuscitation.2399.html.

[27] Roberts BW, Kilgannon JH, Hunter BR, Puskarich MA, Pierce L, Donnino M (2018). Association between early hyperoxia exposure after resuscitation from cardiac arrest and neurological disability: prospective multicenter protocol-directed cohort study. Circulation.

[28] Wang CH, Chang WT, Huang CH, Tsai MS, Yu PH, Wang AY (2014). The effect of hyperoxia on survival following adult cardiac arrest: a systematic review and meta-analysis of observational studies. Resuscitation.

[29] Ebner F, Ullen S, Aneman A, Cronberg T, Mattsson N, Friberg H (2019). Associations between partial pressure of oxygen and neurological outcome in out-of-hospital cardiac arrest patients: an explorative analysis of a randomized trial. Crit Care.

[30] Humaloja J, Litonius E, Efendijev I, Folger D, Raj R, Pekkarinen PT (2019). Early hyperoxemia is not associated with cardiac arrest outcome. Resuscitation.

[31] Elmer J, Scutella M, Pullalarevu R, Wang B, Vaghasia N, Trzeciak S (2015). The association between hyperoxia and patient outcomes after cardiac arrest: analysis of a high-resolution database. Intensive Care Med.

[32] Imberti R, Bellinzona G, Riccardi F, Pagani M, Langer M (2003). Cerebral perfusion pressure and cerebral tissue oxygen tension in a patient during cardiopulmonary resuscitation. Intensive Care Med.

[33] Cavus E, Bein B, Dörges V, Stadlbauer KH, Wenzel V, Steinfath M (2006). Brain tissue oxygen pressure and cerebral metabolism in an animal model of cardiac arrest and cardiopulmonary resuscitation. Resuscitation.

[34] Nelskylä A, Skrifvars MB, Ångerman S, Nurmi J (2022). Incidence of hyperoxia and factors associated with cerebral oxygenation during cardiopulmonary resuscitation. Resuscitation.

[35] Spindelboeck W, Schindler O, Moser A, Hausler F, Wallner S, Strasser C (2013). Increasing arterial oxygen partial pressure during cardiopulmonary resuscitation is associated with improved rates of hospital admission. Resuscitation.

[36] Spindelboeck W, Gemes G, Strasser C, Toescher K, Kores B, Metnitz P (2016). Arterial blood gases during and their dynamic changes after cardiopulmonary resuscitation: a prospective clinical study. Resuscitation.

[37] Rosenthal G, Hemphill JC, Sorani M, Martin C, Morabito D, Obrist WD (2008). Brain tissue oxygen tension is more indicative of oxygen diffusion than oxygen delivery and metabolism in patients with traumatic brain injury. Crit Care Med.

[38] Llitjos JF, Mira JP, Duranteau J, Cariou A (2016). Hyperoxia toxicity after cardiac arrest: what is the evidence?. Ann Intensive Care.

[39] Mackle D, Bellomo R, Bailey M, Beasley R, Deane A, Eastwood G (2020). Conservative oxygen therapy during mechanical ventilation in the ICU. N Engl J Med.

[40] Gelissen H, de Grooth HJ, Smulders Y, Wils EJ, de Ruijter W, Vink R (2021). Effect of low-normal vs high-normal oxygenation targets on organ dysfunction in critically Ill patients: a randomized clinical trial. JAMA.

[41] Semler MW, Casey JD, Lloyd BD, Hastings PG, Hays MA, Stollings JL (2022). Oxygen-saturation targets for critically ill adults receiving mechanical ventilation. N Engl J Med.

[42] Schjørring OL, Klitgaard TL, Perner A, Wetterslev J, Lange T, Siegemund M (2021). Lower or higher oxygenation targets for acute hypoxemic respiratory failure. N Engl J Med.

[43] Robba C, Badenes R, Battaglini D, Ball L, Sanfilippo F, Brunetti I (2022). Oxygen targets and 6-month outcome after out of hospital cardiac arrest: a pre-planned sub-analysis of the targeted hypothermia versus targeted normothermia after Cut-off-hospital cardiac arrest (TTM2) trial. Crit Care.

[44] Nielsen N, Skrifvars MB (2022). Oxygenation and blood-pressure targets in the ICU after cardiac arrest—one step forward. N Engl J Med.

[45] Young P, Bailey M, Bellomo R, Bernard S, Dicker B, Freebairn R (2014). HyperOxic therapy OR NormOxic therapy after out-of-hospital cardiac arrest (HOT OR NOT): a randomised controlled feasibility trial. Resuscitation.

