# Supplementary material for: Hyperoxemia after reperfusion in cardiac arrest patients: a potential dose–response association with 30-day survival
Source: Crit Care. 2023 Mar 6;27:86. doi: 10.1186/s13054-023-04379-9 (PMC9990272; doi:10.1186/s13054-023-04379-9)
Supplement: Supplementary file 1 — Additional file 1. Supplementary Figure 1. Flow of OHCA patients. [file 13054_2023_4379_MOESM1_ESM.pptx]

## Slide 1
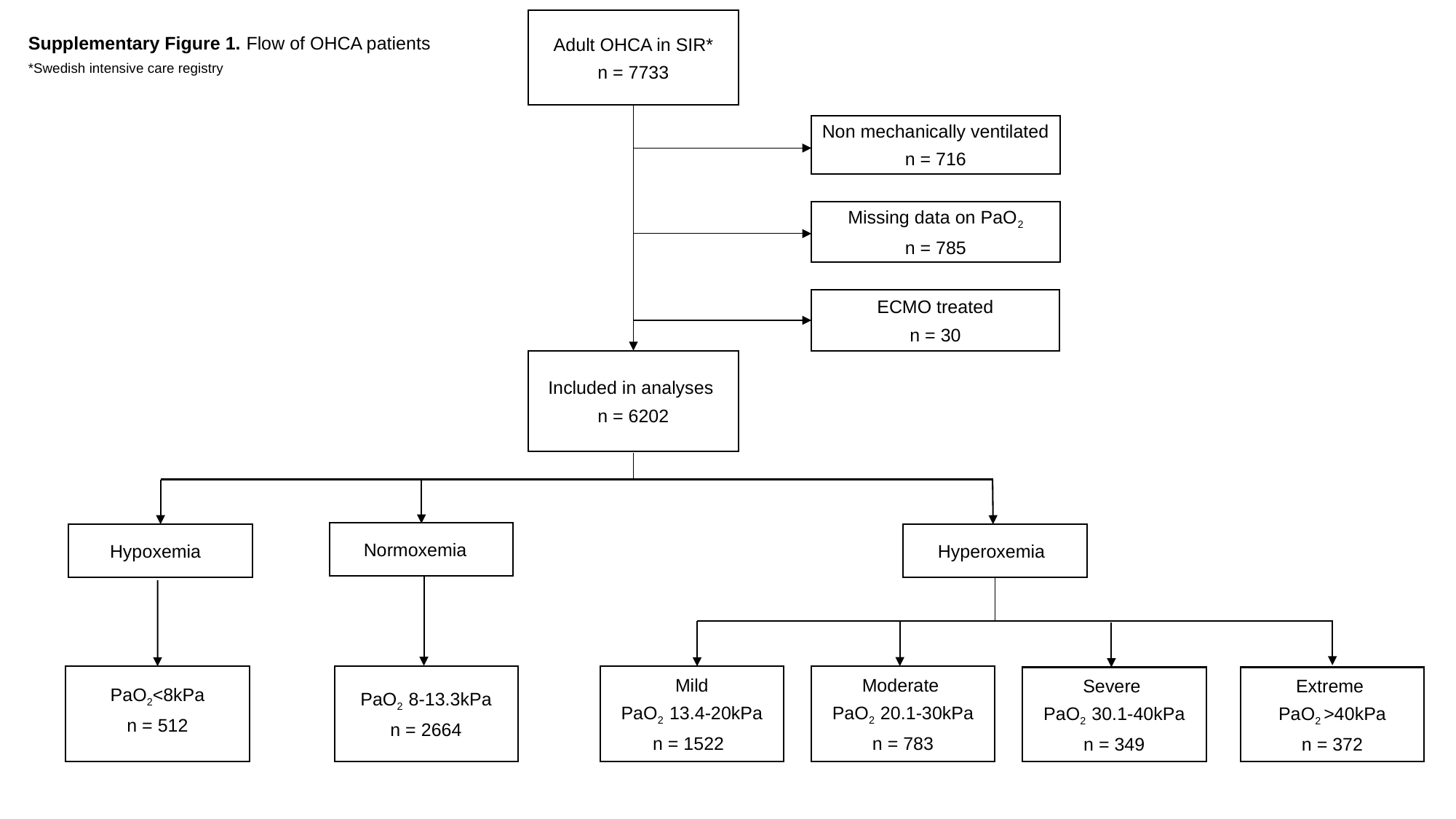

Adult OHCA in SIR*
n = 7733
Supplementary Figure 1. Flow of OHCA patients
*Swedish intensive care registry
Non mechanically ventilated
n = 716
Missing data on PaO2
n = 785
ECMO treated
n = 30
Included in analyses
n = 6202
Normoxemia
Hypoxemia
Hyperoxemia
PaO2 8-13.3kPa
n = 2664
Mild
PaO2 13.4-20kPa
 n = 1522
Moderate
PaO2 20.1-30kPa
n = 783
PaO2<8kPa
n = 512
Severe
PaO2 30.1-40kPa
n = 349
Extreme
PaO2 >40kPa
n = 372
