# Supplementary material for: Hyperoxemia after reperfusion in cardiac arrest patients: a potential dose–response association with 30-day survival
Source: Crit Care. 2023 Mar 6;27:86. doi: 10.1186/s13054-023-04379-9 (PMC9990272; doi:10.1186/s13054-023-04379-9)
Supplement: Supplementary file 2 — Additional file 2. Supplementary Figure 2. Flow of IHCA patients. [file 13054_2023_4379_MOESM2_ESM.pptx]

## Slide 1
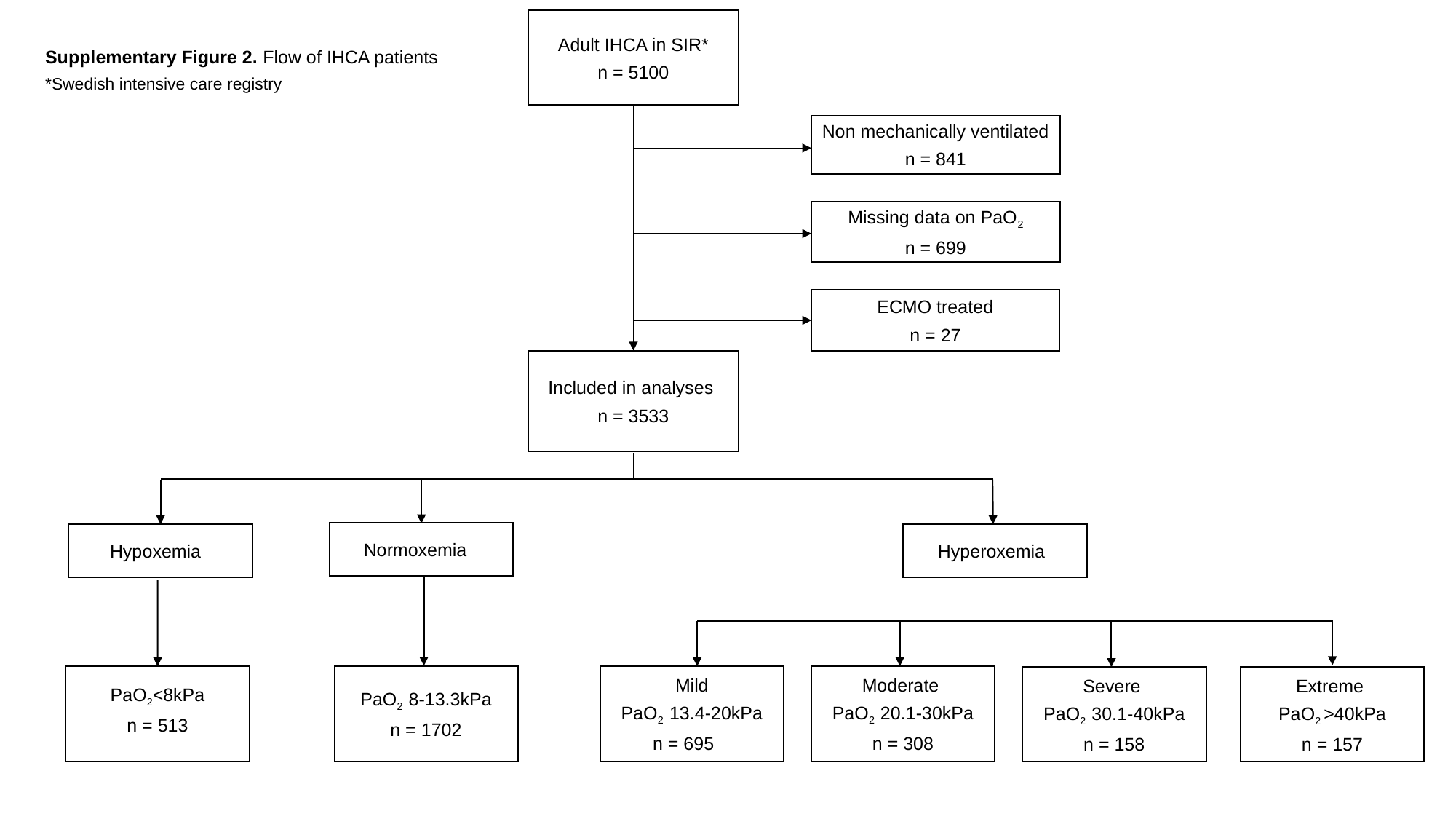

Adult IHCA in SIR*
n = 5100
Supplementary Figure 2. Flow of IHCA patients
*Swedish intensive care registry
Non mechanically ventilated
n = 841
Missing data on PaO2
n = 699
ECMO treated
n = 27
Included in analyses
n = 3533
Normoxemia
Hypoxemia
Hyperoxemia
PaO2 8-13.3kPa
n = 1702
Mild
PaO2 13.4-20kPa
 n = 695
Moderate
PaO2 20.1-30kPa
n = 308
PaO2<8kPa
n = 513
Severe
PaO2 30.1-40kPa
n = 158
Extreme
PaO2 >40kPa
n = 157
