# Supplementary material for: Hyperoxemia after reperfusion in cardiac arrest patients: a potential dose–response association with 30-day survival
Source: Crit Care. 2023 Mar 6;27:86. doi: 10.1186/s13054-023-04379-9 (PMC9990272; doi:10.1186/s13054-023-04379-9)
Supplement: Supplementary file 3 — Additional file 3. Supplementary Figure 3. Adjusted RR for 30-day survival in complete cases. [file 13054_2023_4379_MOESM3_ESM.docx]

**Supplementary figure 3.** Adjusted RR for 30-day survival in complete cases

Adjusted for sex, age, witnessed status, bystander CPR, location, EMS/Rapid response team-response time, initial rhythm, Charlson comorbidity index, SAPS 3 score.
