# Supplementary material for: Hyperoxemia after reperfusion in cardiac arrest patients: a potential dose–response association with 30-day survival
Source: Crit Care. 2023 Mar 6;27:86. doi: 10.1186/s13054-023-04379-9 (PMC9990272; doi:10.1186/s13054-023-04379-9)
Supplement: Supplementary file 4 — Additional file 4. Supplementary Figure 4. Adjusted RR for 30-day survival in patients with shockable rhythms. [file 13054_2023_4379_MOESM4_ESM.docx]

**Supplementary figure 4.** Adjusted RR for 30-day survival in patients with shockable rhythms

Adjusted for sex, age, witnessed status, bystander CPR, location, EMS/Rapid response team-response time, initial rhythm, Charlson comorbidity index, SAPS 3 score.
