# Supplementary material for: Hyperoxemia after reperfusion in cardiac arrest patients: a potential dose–response association with 30-day survival
Source: Crit Care. 2023 Mar 6;27:86. doi: 10.1186/s13054-023-04379-9 (PMC9990272; doi:10.1186/s13054-023-04379-9)
Supplement: Supplementary file 6 — Additional file 6. Supplementary Figure 6. Distribution of patients based on PaO2 [file 13054_2023_4379_MOESM6_ESM.docx]

**Supplementary figure 6.** Distribution of patients based on PaO_2_

__
