# Supplementary material for: Hyperoxemia after reperfusion in cardiac arrest patients: a potential dose–response association with 30-day survival
Source: Crit Care. 2023 Mar 6;27:86. doi: 10.1186/s13054-023-04379-9 (PMC9990272; doi:10.1186/s13054-023-04379-9)
Supplement: Supplementary file 7 — Additional file 7. Supplementary Figure 7. Kaplan-Meier survival analysis. [file 13054_2023_4379_MOESM7_ESM.docx]

**Supplementary figure 7.** Kaplan-Meier survival analysis
